# Self-assembled pagoda-like nanostructure-induced vertically stacked split-ring resonators for polarization-sensitive dichroic responses

**DOI:** 10.1186/s40580-022-00331-9

**Published:** 2022-09-07

**Authors:** Sanghoon Kim, Chunghwan Jung, Jungho Mun, Mooseong Kim, Hyeongkeon Yoon, Junho Jang, Myeongcheol Go, Jaeyong Lee, Junsuk Rho, Jin Kon Kim

**Affiliations:** 1 National Creative Research Initiative Center for Hybrid Nano Materials By High-Level Architectural Design of Block Copolymer, Department of Chemical Engineering, Pohang University of Science and Technology (POSTECH), Pohang, 37673 Republic of Korea; 2 Department of Chemical Engineering, Pohang University of Science and Technology (POSTECH), Pohang, 37673 Republic of Korea; 3 Department of Mechanical Engineering, Pohang University of Science and Technology (POSTECH), Pohang, 37673 Republic of Korea; 4 POSCO-POSTECH-RIST Convergence Research Center for Flat Optics and Metaphotonics, Pohang, 37673 Republic of Korea

**Keywords:** Polarization-sensitive metamaterial, Large-scale fabrication, Anodized aluminum oxide, Block copolymer, Oblique angle deposition, Impedance matching

## Abstract

Stacked split-ring resonators (SSRR) arrays exhibiting polarization-sensitive dichroic responses in both visible and near-infrared wavelengths are realized over a centimeter-scale large area. The SSRR arrays are derived from pagoda-like nanorods fabricated from the self-assembly of a lamellae-forming polystyrene-*b*-poly (methyl methacrylate) copolymer (PS-*b*-PMMA) confined in cylindrical pores of anodized aluminum oxide (AAO) template. Along the nanorod direction, PS and PMMA nanodomains were alternately stacked with the same distance. Silver crescents and semi-hemispherical covers, which are essential for SSRR with the polarization sensitivity, were obliquely deposited on the single side of the nanorod after removing the AAO template and reactive-ion etching treatment. These sophisticated nanoscale architectures made by bottom-up fabrication can be applied to structural color, optical anti-counterfeiting, and commercial optical components in a large area.

**Supplementary Information:**

The online version contains supplementary material available at 10.1186/s40580-022-00331-9.

## Introduction

The concept of the control over light has been expanded such that intriguing applications including negative refraction [1, 2], superlensing [3], and invisibility cloaking [4] are extensively investigated. However, no natural materials have the required electromagnetic properties, such as strong artificial chirality [5–8], hyperbolic dispersion [9], negative magnetic permeability [10], and negative refractive index [11]. To achieve such extraordinary electromagnetic properties, plasmonic metamaterials, or the artificially manufactured metallic structures with unit structures much smaller than the wavelength of light, have been actively researched [12–14]. Negative refractive index has been reported from stacked crescent-shaped structures in the broadband wavelength regime [15], and from two-dimensional perforated metal-dielectric stacks with a thickness much larger than the free-space wavelength [16]. In addition, strong chirality that allows transmission of specific circularly polarized light has been reported from chiral spiral structures [17].

However, most of the reported studies on metamaterials have relied on a top-down approach using traditional lithographic technologies, which are unsuitable for large-scale production of three-dimensional (3D) metamaterials because of their expensive process cost. Although unconventional top-down approaches such as aerosol-jet nanoprinting [18] and two-photon lithography [19, 20] have been attempted to fabricate 3D nanostructures, the resolution limitation and low-throughput issues still remain. Therefore, novel fabrication schemes that can produce geometrically complex plasmonic nanostructures in a large area are in demand.

Self-assembly of block copolymers (BCP) is known to provide various periodic nanostructures with feature sizes of 10–100 nm such as spheres, cylinders, lamellae, and gyroids depending on the volume fraction of the blocks [21, 22]. Nanostructures with more complicated shapes such as concentric rings [23, 24], spirals [25, 26], helices [27, 28], and toroids [29], especially not observed in the bulk state, can be prepared by confining BCP nanodomains in nanospace such as anodized aluminum oxide (AAO) with various pore sizes and architectures [30]. Thus, the self-assembly of block copolymers might be a practical approach to plasmonic metamaterials with complicated shapes over large area and feature sizes below 100 nm that have active in visible and NIR wavelength ranges [31–33].

In this study, we propose large-scale fabrication of stacked split-ring resonators (SSRR) which exhibit polarization-dependent reflections in the visible and infrared wavelengths. For the fabrication of SSRR, we fabricated “pagoda-like” nanostructures in a large area (more than 3 cm × 3 cm) by performing oxygen reactive ion etching (RIE) on stacked lamellar nanodomains of PS*-b-*PMMA prepared by confining to a cylindrical cavity of AAO template. Because the etching rate of PMMA is larger than that of PS [34], the diameter of the PMMA nanodomains in the nanorod becomes smaller than that of PS nanodomains. Thus, original nanorods with the same radius before RIE etching become diameter-modulated nanorods. Finally, after silver (Ag) was selectively deposited only on PS nanodomains by the oblique angle deposition (OAD) [35, 36], SSRR arrays are fabricated. The reflectance spectra of SSRR show experimentally polarization-sensitive dichroic response and are confirmed by finite-difference time-domain (FDTD) simulations. Effective optical parameters of SSRR suggest that the Ag single-sided crescent and semi-hemispherical (quarter spherical) covers generate polarization-dependent plasmonic resonance and cause impedance matching at the visible wavelength. These SSRR made in a suitable way for large-scale fabrication could contribute to the development of metamaterials for the optical anti-counterfeiting label [20, 37] and complex optical elements [33, 38, 39].

## Materials and methods

### Materials and AAO template fabrication

Lamellae-forming PS-*b*-PMMA (SML-98) and PS-*ran*-PMMA-OH were purchased from Polymer Source Inc. and used as received (Table 1). Epoxy resin consisting of 50/45/5 (wt%) Araldite resins (grade 6005)/dodecenylsuccinic anhydride/DMP-30 was purchased from Polysciences Inc. The well-known two-step anodization procedure was used to create the AAO template with the hexagonal close-packed pore structure over a large area [40–44]. The first anodization process was carried out with 0.3 M aqueous oxalic acid (C_2_H_2_O_4_) solution. After the disordered pores in the AAO template were removed, the second anodization step was performed for 120 s. The interpore distance (*D*_*int*_) between two neighboring pores of the AAO template was 100 nm. After pore widening for 30 min in a 0.1 M H_3_PO_4_ aqueous solution at 30 °C, the final pore diameter (*D*) was 50 nm. Detailed information of anodization and etching steps for preparing AAO templates is described in Additional file 1: Fig. S1.

**Table 1:** Molecular characteristics of PS*-b-*PMMA and PS-*ran*-PMMA employed in this study

Polymer	Lot No (Supplier)	M_n_ [kg·mol^−1^]	*Đ*^a^	*L*_o_^b^ [nm]	ƒ_PS_^c^
SML-98	P2355 (Polymer source)	98	1.13	47.3	0.54
PS-ran-PMMA-OH	P6469A (Polymer source)	7	1.48		0.57

^a^Polydispersity index (Mw/Mn, M_n_ and M_w_ are the number and weight average molecular weights, respectively)

^b^The lamellar domain spacing measured by small-angle X-ray scattering

^c^Volume fraction of the PS block measured by ^1^H NMR and the known densities of PS (1.04 g·cm^−3^) and PMMA (1.18 g·cm^−3^)

### Optical modeling and simulations

**Fig. 1:**
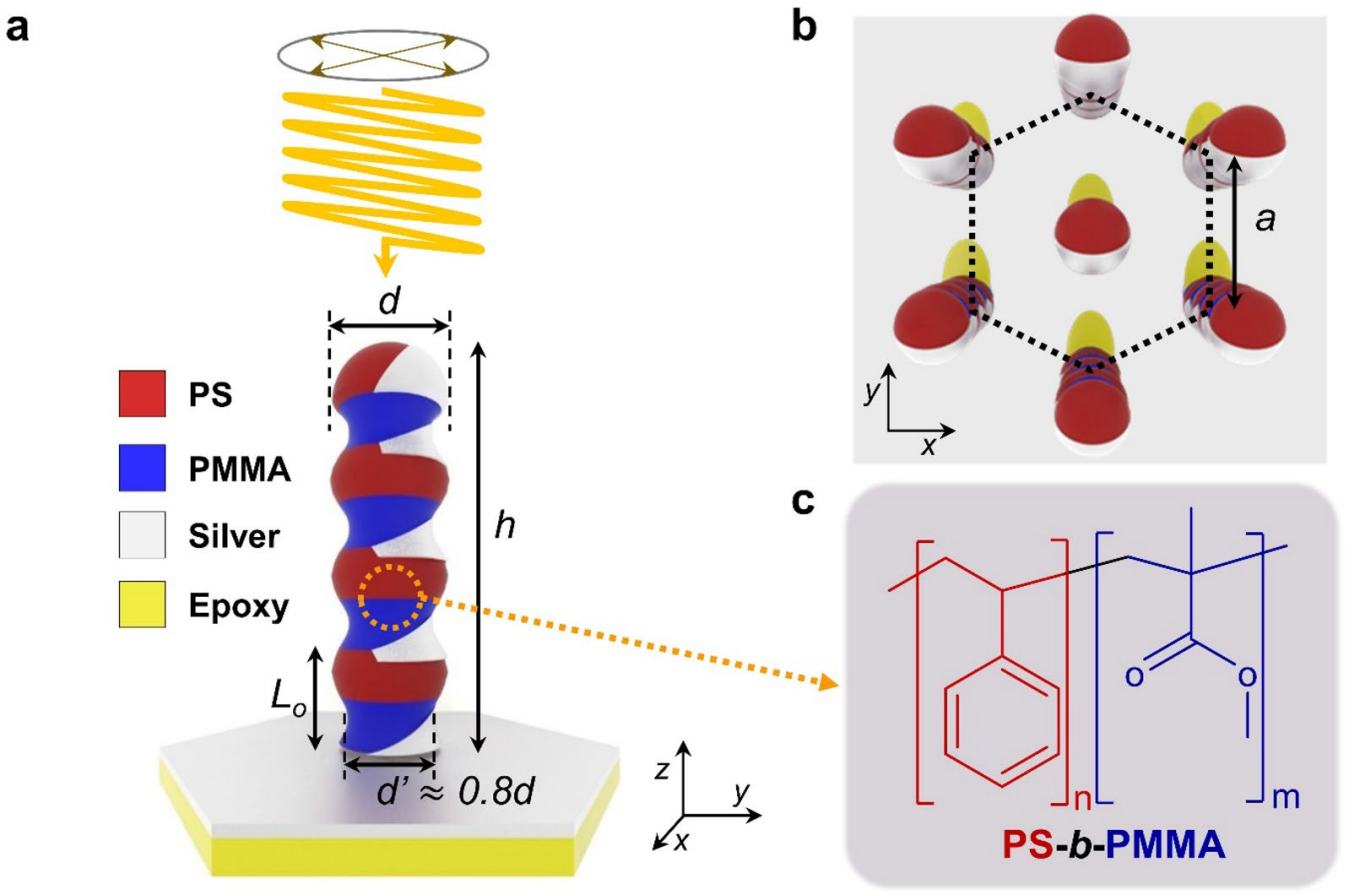
Schematic of stacked split-ring resonators (SSRR). **a** Tilted view of a SSRR and geometric parameters (d and d’ the diameter of PS and PMMA nanodomains, respectively, h: the height of nanorod). **b** Top-view of a hexagonal array of SSRR (a: the center-to-center distance between two neighboring nanorods). **c** Chemical structures of PS*-b-*PMMA

Electromagnetic properties of SSRRs were calculated using the three-dimensional full-wave numerical simulation with a commercial finite-difference time-domain (FDTD) solver (Lumerical Inc). The hexagonally packed SSRR was designed in the form of regular-spaced Ag crescents stacked on only a single side of PS-*b*-PMMA nanorods. The diameter *d* of PS domain is 50 nm, the diameter of PMMA domain (*d*’) is about 0.8*d*, the height *h* of nanorod is 200 nm, and the center-to-center distance between nanorods *a* is 100 nm. The y-axis is defined by the Ag deposition direction, and the x-axis is the vertical direction of the y-axis (Fig. 1). The size and shape of PS-*b*-PMMA nanorods were essentially the same as parameters (*D, H,* and *D*_*in*t_) of pores in the fabricated AAO templates. The thickness and shape of Ag crescents were determined by the oblique angle deposition process. Specific geometric parameters were obtained by SEM measurements. The refractive index of epoxy resin of 1.53 obtained by ellipsometry was used for simulation, and the optical parameters of Ag were taken from Johnson and Christy [45]. The elements of S-parameter matrix were obtained through the built-in function of the FDTD solution. The optical properties of the metamaterials, such as refractive index, permittivity, permeability, and optical impedance, were obtained by the S-parameter retrieval methods using Matlab.

### Fabrication of stacked split-ring resonators (SSRR)

**Fig. 2:**
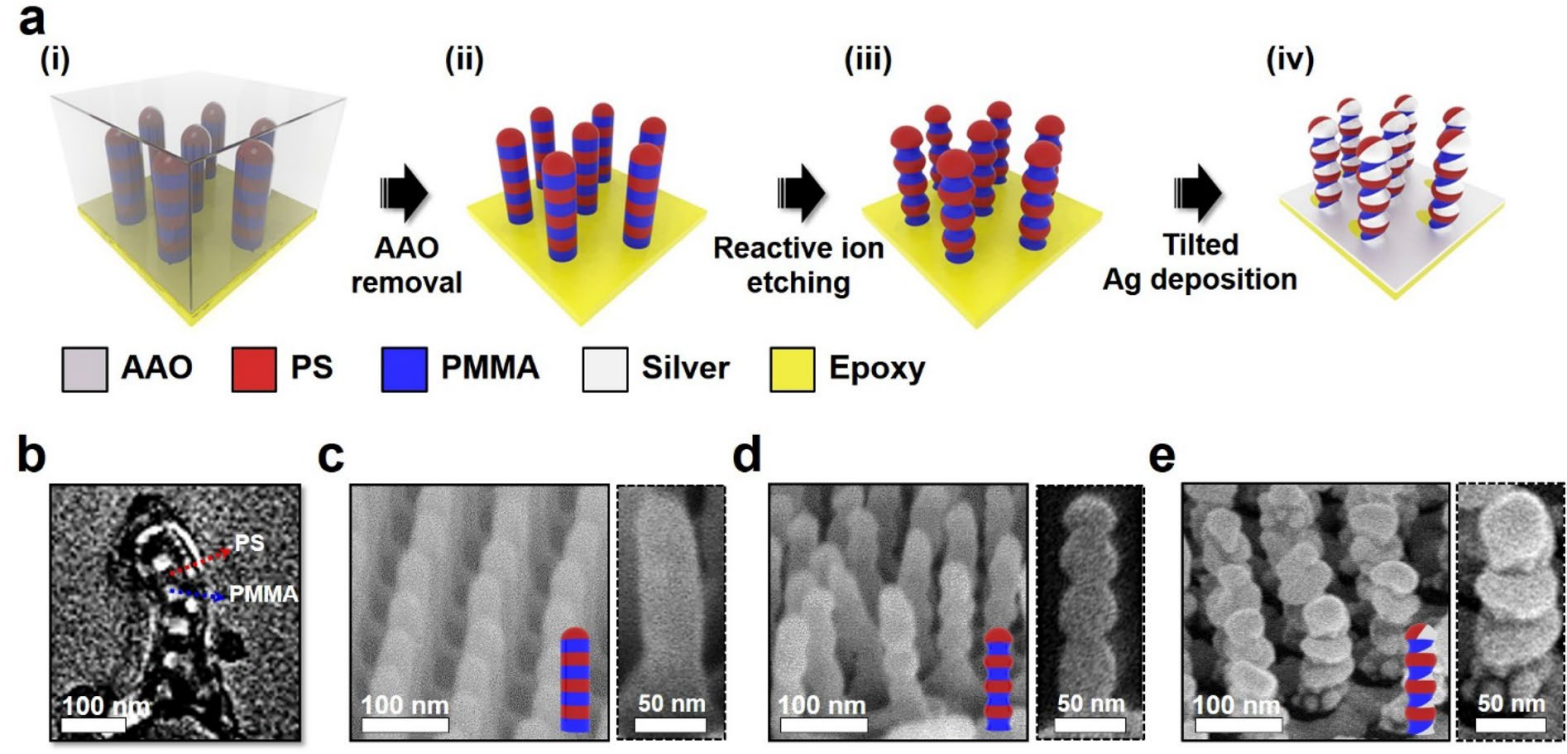
Fabrication of a high-density array of stacked split-ring resonators (SSRR). **a** Schematic of fabrication of SSRR nanostructures. TEM image of **b** stacked lamellae. FE-SEM images of **c** the stacked lamellae nanorods, **d** pagoda-like nanostructures, and **e** stacked split-ring resonators (the brighter part is Ag and the darker part is PMMA block). Dotted boxes in **c**–**e** are expanded images

Fabrication of a high-density array of SSRR is schematically shown in Fig. 2a. First, the AAO template was spin-coated with PS-*ran*-PMMA-OH solution in toluene (1 wt%) and annealed for 3 days at 180 °C under vacuum so that the walls of AAO nanopores were neutral for both PS and PMMA chains. The unreacted PS-*ran*-PMMA-OH chains were eliminated by sonication in toluene. Then, SML-98 was dissolved in toluene (2 wt%) and spin-coated onto the AAO template, and annealed at 200 °C for 3 days in a vacuum, followed by quenching to room temperature. The as-annealed AAO template was coated with epoxy resin (with a thickness of 0.5 mm), which was then cured for 12 h at 60 °C (step i). Next, the aluminum layer attached to AAO template was removed by using a CuCl_2_ aqueous solution, and the AAO template was removed by immersing in a 0.4 M sodium hydroxide (NaOH) aqueous solution for 90 min, followed by rinsing with DI water. To maintain the vertically aligned and free-standing PS*-b-*PMMA nanorods array on the epoxy resin substrate, the water was completely removed by freeze-drying [46]. (step ii). A high-density array of pagoda-like nanorods was prepared by O_2_ RIE treatment (30 W and 60 s) (step iii). Finally, 10-nm-thick Ag was selectively deposited on the PS nanodomains of the nanorods from thermal evaporation with 30° tilt angle at a rate of 0.1 nm·s^−1^ under high vacuum to form SSRR (step iv) [47].

### Characterization

The cross-sectional image of the PS-b-PMMA nanodomains confined in cylindrical nanopores of the AAO template was observed by transmission electron microscopy (TEM; S-7600: Hitachi Ltd., operating at an accelerating voltage of 80 kV). We also confirmed the architectures of PS-*b*-PMMA nanorods on the epoxy layer sequentially after reactive ion etching and oblique angle deposition via field emission scanning electron microscopy (FE-SEM; S-4800: Hitachi Ltd., operating at 3 kV) with energy dispersive spectroscopy (EDS). Reflectance spectra were measured via a Fourier transform-infrared spectrometer (Bruker Vertex 70) equipped with a microscope (Bruker Hyperion 2000).

## Results and discussions

In the previous work, we obtained stacked lamellae of PS-*b*-PMMA by confinement in AAO cylindrical cavity with various diameters when AAO surface was treated by a neutral brush [48]. Figure 2b gives a TEM image of SML-98 confined in nanopore of AAO template with a diameter of 50 nm after the wall of the AAO membrane was treated by a neutral brush of PS-*ran*-PMMA-OH. PS nanodomain stained by RuO_4_ is represented by the dark area. The TEM image showed that each PS and PMMA nanodomain was alternatively stacked along the direction of the nanorod. The number of stacks is controlled by the height (*H*) of the AAO template and the lamellar domain spacing (*L*_o_) of PS-*b*-PMMA. A high-density array of vertically aligned nanorods was successfully fabricated over a large area (3 × 3 cm^2^) by removing the AAO template (Fig. 2c). The straight cylindrical nanorods with a diameter of 50 nm are confirmed by SEM (inset of Fig. 2c). When O_2_ plasma treatment was performed, we obtained pagoda-like nanorods where the diameter (~ 40 nm) of PMMA nanodomains was smaller than that (50 nm) of PS nanodomains because of the higher etching rate of PMMA than PS. But, the distance (47.0 nm) between each PS nanodomains remained as *L*_o_ of SML-98 (47.3 nm) (Fig. 2d). By utilizing the wavy surface of these pagoda-like nanorods, we selectively deposited Ag on PS nanodomains with a tilted angle of 30°. In this situation, Ag could not be deposited on PS nanodomain in the shadow side of the nanorods, three stacked crescent-shaped nanorings, and a semi-hemispherical cover in the top of the nanorod were obtained (Fig. 2e and Additional file 1: Fig. S2). This is because of *H/L*_*o*_ ~ 4. Therefore, we successfully prepared a high-density array of SSRR nanorods over a large area (Additional file 1: Fig. S3).

**Fig. 3:**
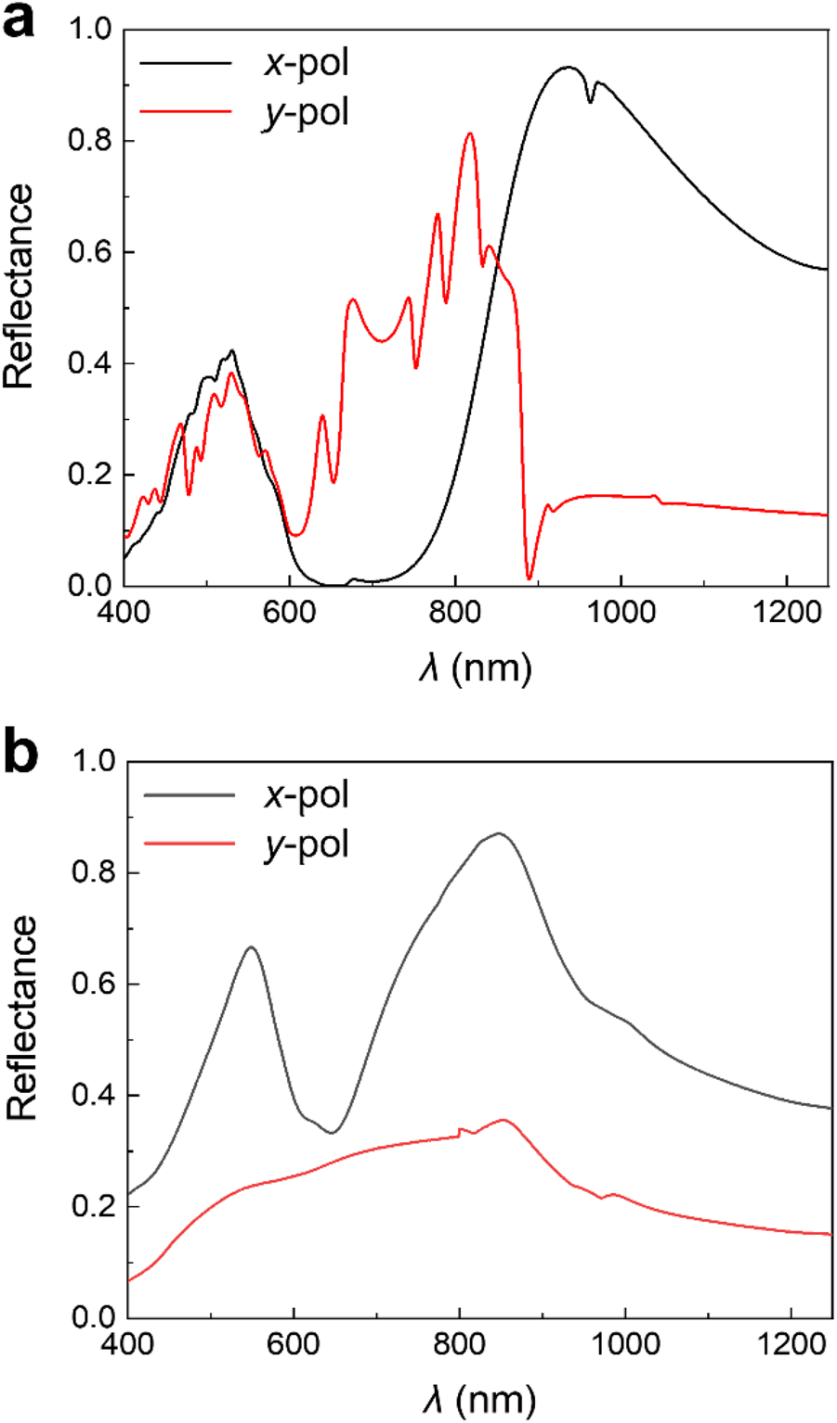
Reflectance spectra of SSRR under x- and y- polarized lights. **a** FDTD simulation. **b** Experiment

The SSRR shows different reflectance spectra depending on the polarization angles of incident lights, because of its anisotropic shape of the plasmonic crescents and semi-hemispherical cover. The SSRR metamaterials are designed to act as a polarization-sensitive dichroic mirror, which selectively reflects specific polarized light in two different wavelength ranges (Fig. 3a). The suggested SSRR shows a significantly different band reflection depending on the incident polarization, and the reflection bands of *x*- and *y*- polarization intersect at 850 nm. In detail, the *x*- and *y*- polarized lights predominantly reflected at 850–1250 and 600–850 nm respectively. This polarization dependency is confirmed in the experimentally measured reflectance spectra (Fig. 3b). However, several inevitable fabrication errors generate a discrepancy between the simulation and experiment. The uneven deposition of the plasmonic ring resonators could form random nano-dot shapes instead of continuous layers. The discrete Ag islands result the blue shift of reflectance at *x*-polarized incidence and the drop of reflectance at *y*-polarized incidence (Additional file 1: Fig. S4). The bending and tilting of the polymer nanorods due to the high aspect ratio also provoke the mismatch of reflectance. The increased area of exposed Ag due to the deformation of nanorods may increase the reflectance under *x*-polarized light, especially at 400–500 nm (Additional file 1: Fig. S5).

**Fig. 4:**
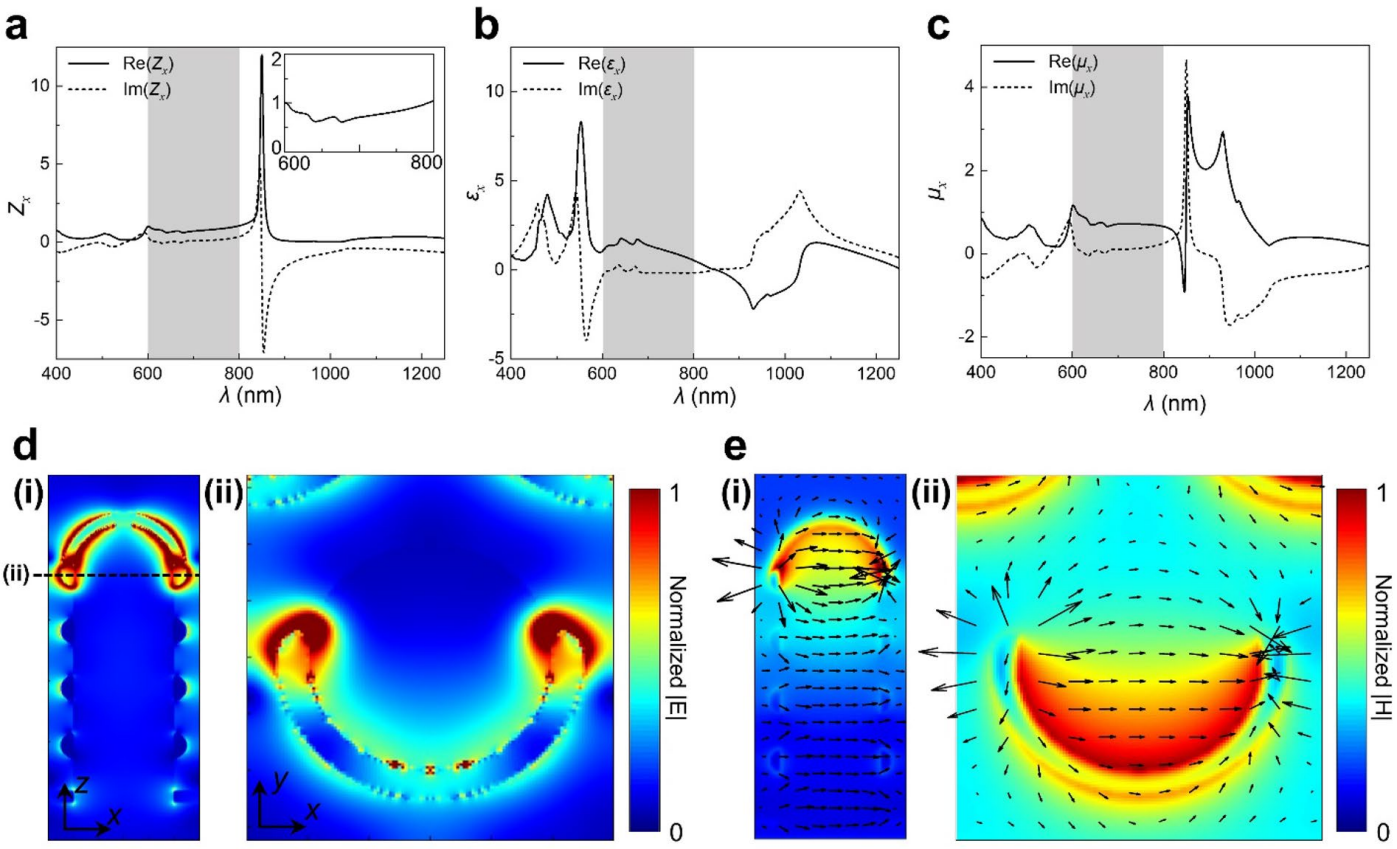
Effective optical parameters and electrical field diagrams of SSRR under *x*-polarized light. Effective **a** wave impedance, **b** permittivity, and **c** permeability of SSRR. **d** Electric and **e** magnetic field profile of SSRR at λ = 550 nm and 837 nm, respectively. (i) *x*–*z* cross-section and (ii) *x*–*y* cross-section of SSRR

To clarify the optical responses of SSRR, effective electromagnetic parameters have been calculated based on S-parameter retrieval method. The analysis in the main text was performed for *x*-pol, and the calculation results of the effective optical parameter of *y*-pol are shown in Additional file 1: Fig. S6. The significantly lower reflection in 600–800 nm at *x*-polarized light incidence is explained by the impedance matching between air and the metamaterial layer (Fig. 4a). The effective wave impedance is defined as the square root of the ratio of effective permeability to permittivity, and the impedance of unity is achieved when these two optical parameters become equal (Figs. 4b and c). It has been known that most of the incident light passes through the metamaterials in the impedance matching region. In addition, effective optical parameters (Z, ε, and μ) of the SSRR substructure were performed to determine the origins of resonance (Additional file 1: Fig. S7).

In addition, we analyze strong electric and magnetic resonances of SSRR that are generated by the top crescent-shaped structures and semi-hemispherical covers (Figs. 4d and e). The electric resonance that appears with the strong resonance of permittivity at λ = 550 nm comes from the localized plasmonic resonance of Ag, and the accompanied electric field amplification can be confirmed (Fig. 4d). The magnetic resonance that appears with strong resonance in permeability at λ = 837 nm comes from the magnetic field produced by the electric current loop along the surface of the semi-hemispherical cover (Fig. 4e). This analysis is consistent with the simulation result that the optical response hardly changes under different geometric parameters related to the side ring of SSRR; *i.e.* as the number of the stacked ring and the gap distance (Additional file 1: Fig. S8). In contrast, the other geometric parameters, such as period and radius of metamaterial, showed the effect of lowering the polarization dependency or shifting the cut-on wavelength. In particular, it is noteworthy that the polarization sensitivity decreases as the period increases, and the polarization dependence disappears when fabricated with *a* = 500 nm (Additional file 1: Fig. S9).

## Conclusions

We fabricate a novel polarization-sensitive metamaterial by utilizing high-density array of stacked Ag crescents nanorods derived from pagoda-like nanorods via BCP self-assembly. This method provides multi-layers metal nanostructure over a large area, which could not be possible from a top-down approach. High selectivity in linear polarization at the visible and NIR regime was observed, and a similar tendency was confirmed in the experiments, although discrepancy appears due to the inevitable process error in the bottom-up approach. We further analyze electric and magnetic resonances that appear in both the effective optical parameters and the electromagnetic near-field profiles. It was confirmed that the electric resonance was mainly induced by the plasmonic crescent and semi-hemispherical cover located on the top of nanostructures, and the magnetic resonance was induced by the curved semi-hemispherical cover that produces electric field loop and magnetic fields. These plasmonic resonances open up the possibility to engineer the optical responses by changing geometrical parameters, such as the period or radius of the nanorods. The development of polarization-sensitive metamaterials through a bottom-up process would be applicable to optical anti-counterfeiting label and commercial optical components.

## Supplementary Information

**Additional file 1: Figure S1.** Top-view (top, i) and 70° tilted-view (bottom, ii) SEM images of AAO template with various diameters (*D*) and height (*H*) is (a) 30 nm, 200 nm (b) 50 nm, 200 nm and (c) 100 nm, 300 nm. **Figure S2.** FE-SEM images with energy dispersive spectroscopy (EDS) results (a) before and (b) after tilt silver deposition. The EDS peak of silver at around 3.0 keV confirms the presence of silver element. **Figure S3. **(a) A photograph of a sample. (b) Top-view FE-SEM images of a high-density array of nanorods (inset is a expanded image) and (c) diameter of SSRR nanostructure at the four position over a large area (3 × 3 cm^2^). **Figure S4. **Simulated reflection spectrum of SSRRs with (a) uniformly deposited Ag film and (b) island-like discretely deposited Ag (Radius = 20 nm and island spacing distance = 10 nm). Black solid line: Reflectance under *x*-polarized light. Red solid line: Reflectance under *y*-polarized light. Blue dash line: The difference between *x*- and *y*- polarized light. **Figure S5**. Simulated reflection spectrum of (a) vertically erected SSRR and (b) tilted SSRR (Tilt angle = 20°). Black solid line: Reflectance under *x*-polarized light. Red solid line: Reflectance under *y*-polarized light. Blue dash line: The difference between *x*- and *y*- polarized light. **Figure S6. **Effective optical parameters of SSRR under *y*-polarized light. Effective (a) wave impedance, (b) permittivity, and (c) permeability of SSRR. **Figure S7**. Effective optical parameters (Z, ε, and μ) of the SSRR substructure come from S-parameter retrieval method. (a) Schematic image and inclusion relationship table for each substructure. (b) Effective wave impedance, (c) permittivity, and (d) permeability under x-polarized light. **Figure S8. **Effect of (a) the center-to-center distance between nanorods *a, *(b) the nanorod diameter *d*, (c) the number of ring *N*, and (d) neighboring inter-rod distance *L*_*o*_ on the reflectance differences between *x*- and *y*-polarized light (R_x_-R_*y*_). **Figure S9** (a) Schematic image of a hexagonal array of SSRR (a: the center-to-center distance between two neighboring nanorods). (b) Experimentally measured reflectance of the array of SSRR. FE-SEM image of a low-density array of (c) pagoda-like nanorods (d) SSRR with large perodicity (a = 500 nm).

## Data Availability

The datasets used and/or analysed during the current study are available from the corresponding author on reasonable request.
