# Supplementary material for: Self-assembled pagoda-like nanostructure-induced vertically stacked split-ring resonators for polarization-sensitive dichroic responses
Source: Nano Converg. 2022 Sep 7;9:40. doi: 10.1186/s40580-022-00331-9 (PMC9452615; doi:10.1186/s40580-022-00331-9)
Supplement: Supplementary file 1 — Additional file 1: Figure S1. Top-view (top, i) and 70° tilted-view (bottom, ii) SEM images of AAO template with various diameters (D) and height (H) is (a) 30 nm, 200 nm (b) 50 nm, 200 nm and (c) 100 nm, 300 nm. Figure S2. FE-SEM images with energy dispersive spectroscopy (EDS) results (a) before and (b) after tilt silver deposition. The EDS peak of silver at around 3.0 keV confirms the presence of silver element. Figure S3. (a) A photograph of a sample. (b) Top-view FE-SEM images of a high-density array of nanorods (inset is a expanded image) and (c) diameter of SSRR nanostructure at the four position over a large area (3 × 3 cm2). Figure S4. Simulated reflection spectrum of SSRRs with (a) uniformly deposited Ag film and (b) island-like discretely deposited Ag (Radius = 20 nm and island spacing distance = 10 nm). Black solid line: Reflectance under x-polarized light. Red solid line: Reflectance under y-polarized light. Blue dash line: The difference between x- and y- polarized light. Figure S5. Simulated reflection spectrum of (a) vertically erected SSRR and (b) tilted SSRR (Tilt angle = 20°). Black solid line: Reflectance under x-polarized light. Red solid line: Reflectance under y-polarized light. Blue dash line: The difference between x- and y- polarized light. Figure S6. Effective optical parameters of SSRR under y-polarized light. Effective (a) wave impedance, (b) permittivity, and (c) permeability of SSRR. Figure S7. Effective optical parameters (Z, ε, and μ) of the SSRR substructure come from S-parameter retrieval method. (a) Schematic image and inclusion relationship table for each substructure. (b) Effective wave impedance, (c) permittivity, and (d) permeability under x-polarized light. Figure S8. Effect of (a) the center-to-center distance between nanorods a, (b) the nanorod diameter d, (c) the number of ring N, and (d) neighboring inter-rod distance Lo on the reflectance differences between x- and y-polarized light (Rx-Ry). Figure S9 (a) Schematic image [file 40580_2022_331_MOESM1_ESM.docx]

**Self-assembled pagoda-like nanostructure-induced vertically stacked split-ring resonators for polarization-sensitive dichroic responses**

Sanghoon Kim^1,‡^, Chunghwan Jung^2,‡^, Jungho Mun^3,‡^, Mooseong Kim^1^, Hyeongkeon Yoon^1^, Junho Jang^1^, Myeongcheol Go^1^, Jaeyong Lee^1^, Junsuk Rho^2,3,4,*^, and Jin Kon Kim^1,*^

^1^National Creative Research Initiative Center for Hybrid Nano Materials by High-level Architectural Design of Block Copolymer, Department of Chemical Engineering, Pohang University of Science and Technology (POSTECH), Pohang 37673, Republic of Korea

^2^Department of Chemical Engineering, Pohang University of Science and Technology (POSTECH), Pohang 37673, Republic of Korea

^3^Department of Mechanical Engineering, Pohang University of Science and Technology (POSTECH), Pohang 37673, Republic of Korea

^4^POSCO-POSTECH-RIST Convergence Research Center for Flat Optics and Metaphotonics, Pohang 37673, Republic of Korea

[‡] Both are equally contributed.

*Corresponding author: Junsuk Rho and Jin Kon Kim

Tel.: +82-54-279-2187 and +82-54-279-2276

E-mail address: [jsrho@postech.ac.kr](mailto:jsrho@postech.ac.kr) and [jkkim@postech.ac.kr](mailto:jkkim@postech.ac.kr)

**S1. Fabrication of anodized aluminum oxide (AAO) templates with various diameters**

Anodized aluminum oxide (AAO) templates with various pore diameters were fabricated by two-step anodization method. A highly pure aluminum plate (99.999%, 1 mm thickness) was sonicated in acetone for 30 min before being electrochemically polished for 50 min at 7°C and 20 V in a polishing solution (ethanol: perchloric acid (HClO_4_) = 4:1 v/v). The polished aluminum plate was washed with ethanol and deionized (DI) water alternately. The anodization was carried out with 0.3 M oxalic acid (C_2_H_2_O_4_) aqueous solution at 15°C and 70 V for 12 h and was etched by an etchant solution (1.8wt% chromic acid (H_2_CrO_4_) and 6wt% H_3_PO_4_ in deionized water) at 65^o^C for over 6 h, and washed by DI water.

The second anodization was performed with 0.3 M C_2_H_2_O_4_ aqueous solution at 15°C and 70 V for 120 s, generating hexagonally packed cylindrical nanopores with interpore distance (*D_int_*) of 100 nm, pore diameter (*D*) of 20 nm, and height (*H*) of 200 nm. The pores were further enlarged after the wet etching by the immersion of the sample in 0.1 M H_3_PO_4_ aqueous solution at 30°C for 10 min and 30 min, producing *D* = 30 nm and 50 nm, respectively, whereas *H* is fixed at 200 nm, as shown in each Figure S1b and S1c. We also fabricated another AAO template with large periodicity (*D_int_* = 500 nm) by anodizing with 0.1 M H_3_PO_4_ aqueous solution at 0°C and 195 V for 180 s, generating nanopores with a diameter (*D*) of 100 nm and a thickness (*H*) of 300 nm, as shown in Figure S1c.


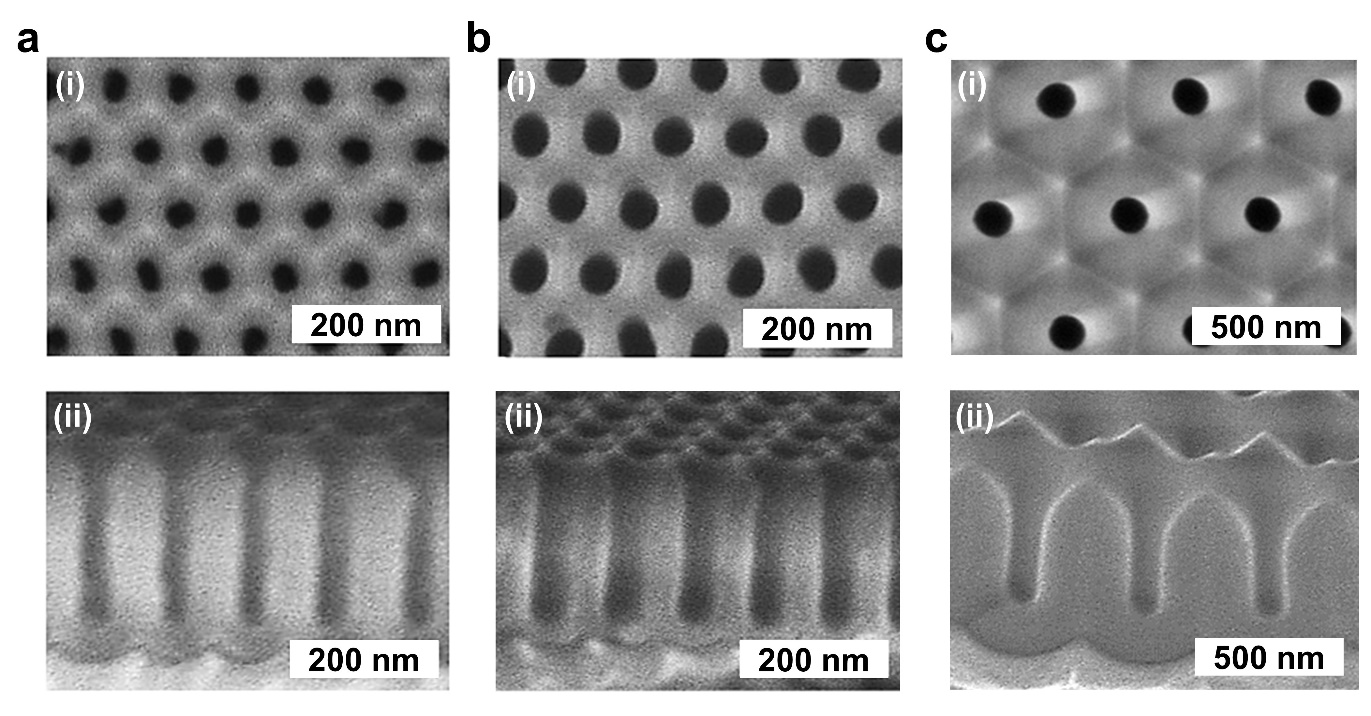


**Figure S1.** Top-view (top, i) and 70° tilted-view (bottom, ii) SEM images of AAO template with various diameters (*D*) and height (*H*) is (a) 30 nm, 200 nm (b) 50 nm, 200 nm and (c) 100 nm, 300 nm.

**S2. Energy dispersive spectroscopy (EDS) analysis of pagoda-like nanorods and SSRR nanostrucrues.**


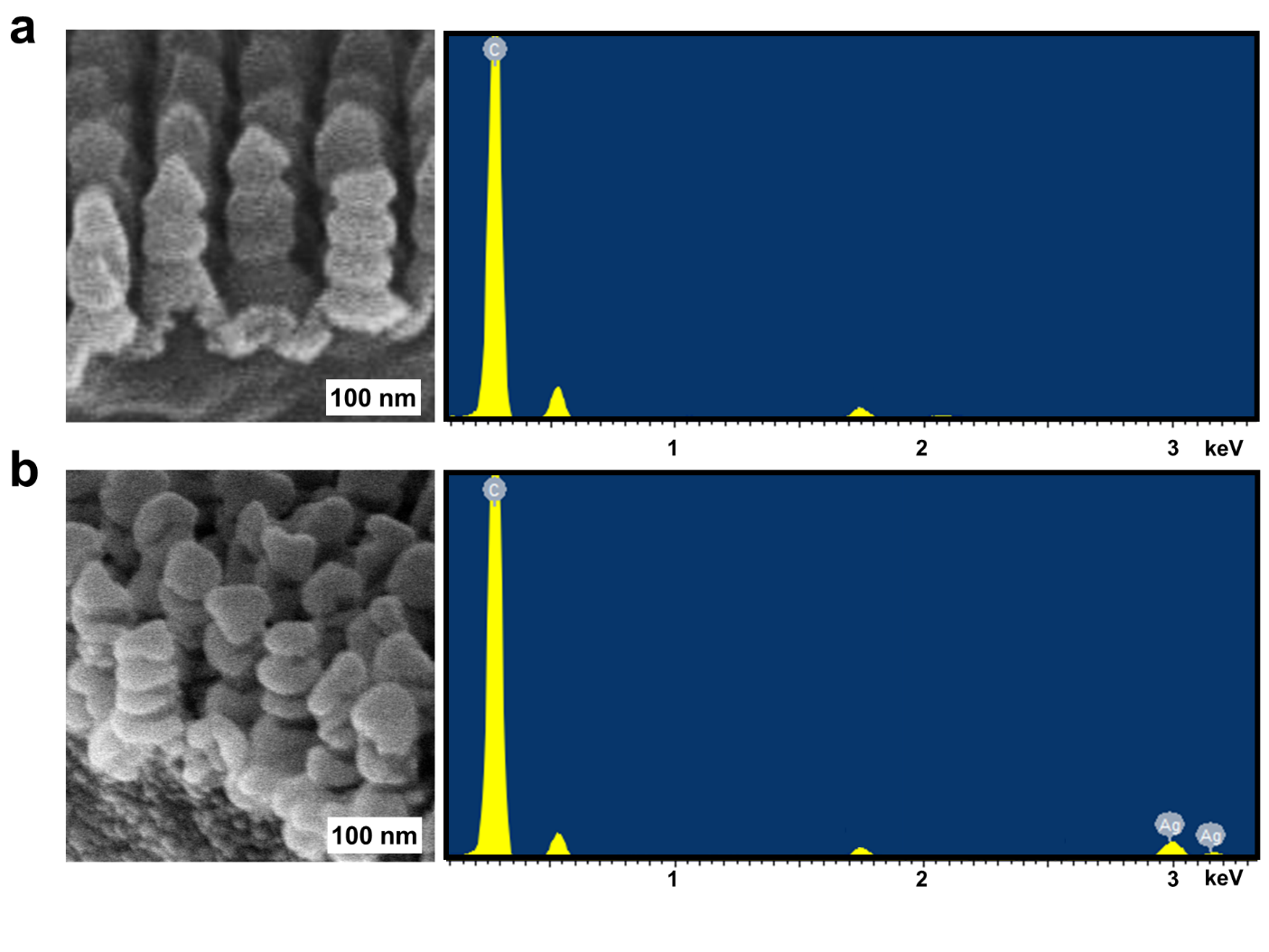


**Figure S2.** FE-SEM images with energy dispersive spectroscopy (EDS) results (a) before and (b) after tilt silver deposition. The EDS peak of silver at around 3.0 keV confirms the presence of silver element.

**S3.** **A** **high-density array of SSRR nanostructures over a large area**

**
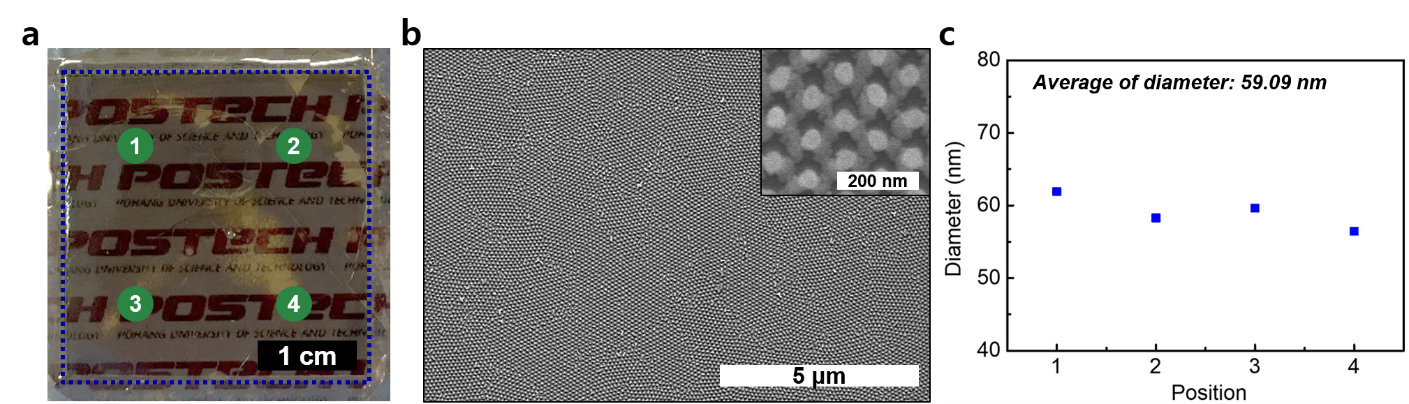
**

**Figure S3.** (a) A photograph of a sample. (b) Top-view FE-SEM images of a high-density array of nanorods (inset is a expanded image) and (c) diameter of SSRR nanostructure at the four position over a large area (3 × 3 cm^2^).

**S4. Morphology effects of Ag film on the polymer nanorods**

**
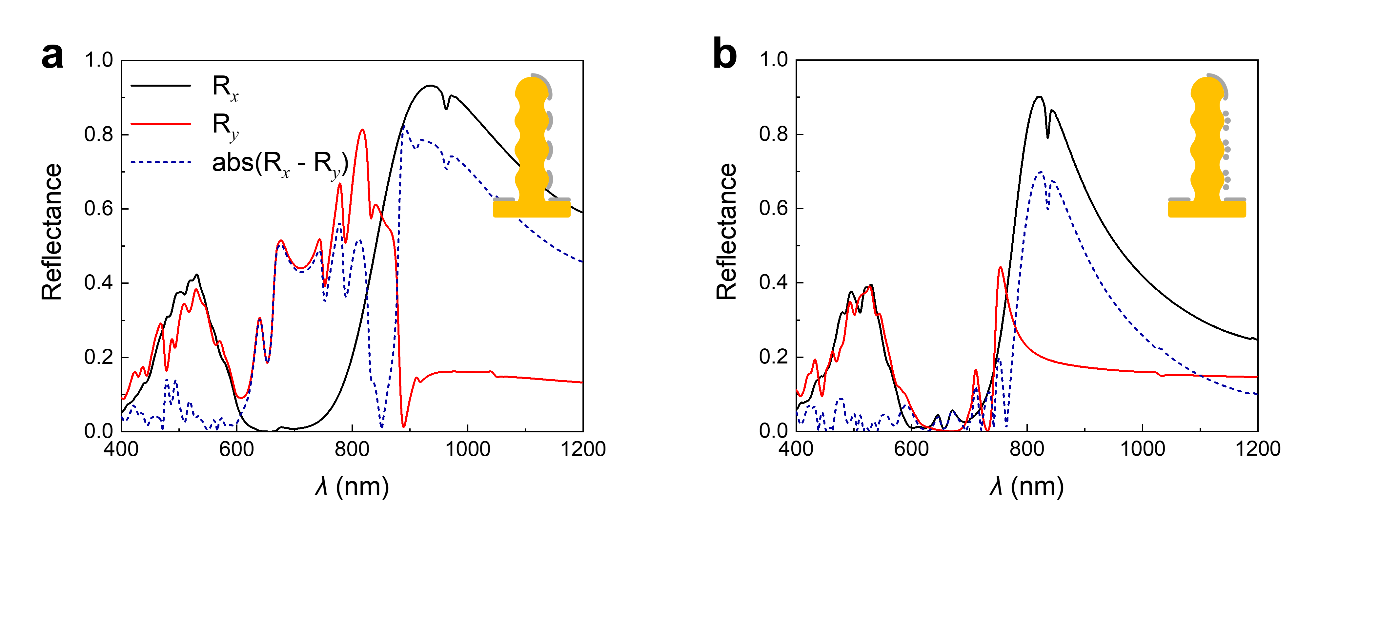
**

**Figure S4.** Simulated reflection spectrum of SSRRs with (a) uniformly deposited Ag film and (b) island-like discretely deposited Ag (Radius = 20 nm and island spacing distance = 10 nm). Black solid line: Reflectance under *x*-polarized light. Red solid line: Reflectance under *y*-polarized light. Blue dash line: The difference between *x*- and *y*- polarized light.

**S5. Tilting effects of the PS-*b*-PMMA nanorods**

**
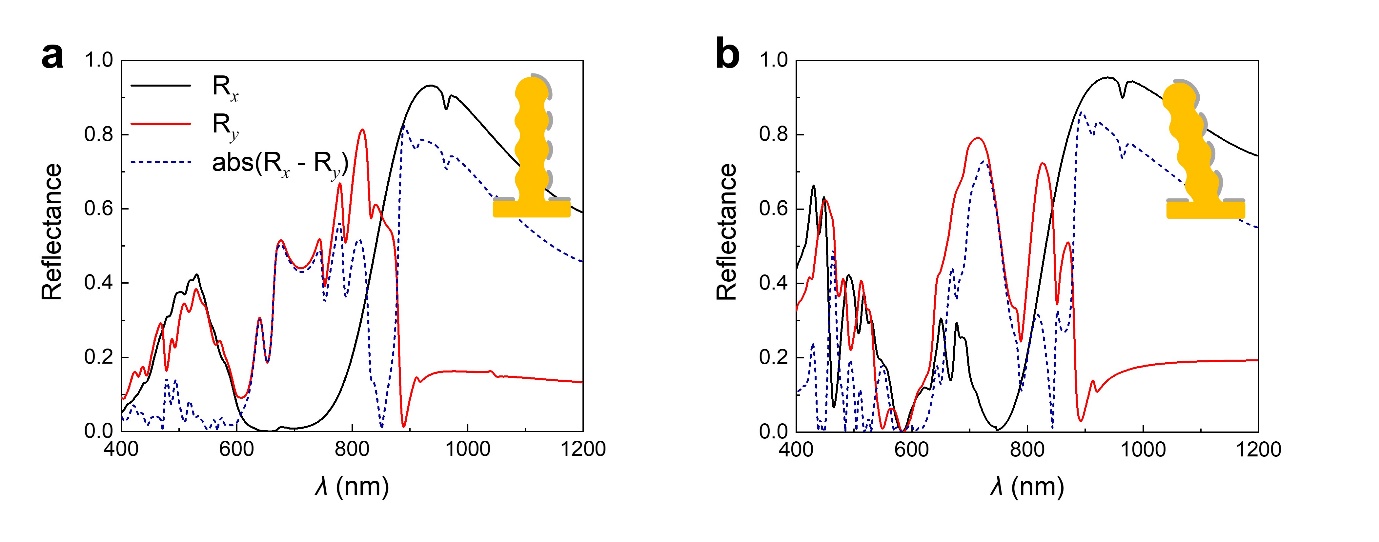
**

**Figure S5**. Simulated reflection spectrum of (a) vertically erected SSRR and (b) tilted SSRR (Tilt angle = 20°). Black solid line: Reflectance under *x*-polarized light. Red solid line: Reflectance under *y*-polarized light. Blue dash line: The difference between *x*- and *y*- polarized light.

**S6. Effective metamaterial parameters under *y*- polarized light**

**
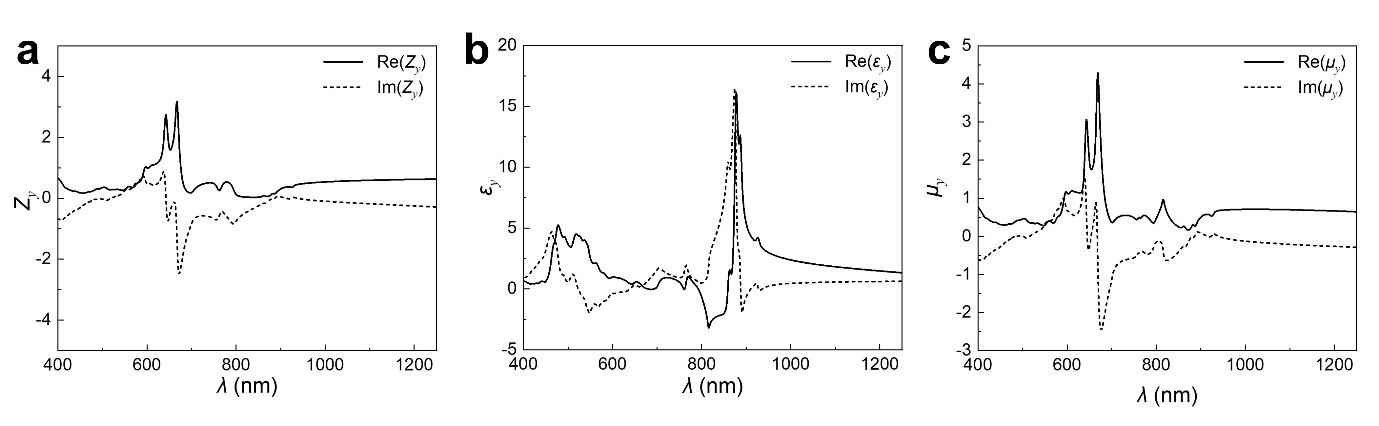
**

**Figure S6.** Effective optical parameters of SSRR under *y*-polarized light. Effective (a) wave impedance, (b) permittivity, and (c) permeability of SSRR.

**S7. Effective optical parameters from various substructures for the origins of resonance**

To understand the role of each part of the complicated geometry, the S-parameter retrieval for different structures was performed. Models 1, 3, 4, and 7 have magnetic (μ) resonance at 800-1000 nm, which corresponds to the response from the hemispherical top layer. This resonance is not observed from models 2, 5, 6, and 8 without the top layer. This resonance is strongly affected by nearby middle silver layers. Models 1, 2, 3, and 5 have electric (ε) resonance at 450-550 nm, which corresponds to the response from the bottom silver layer.

**
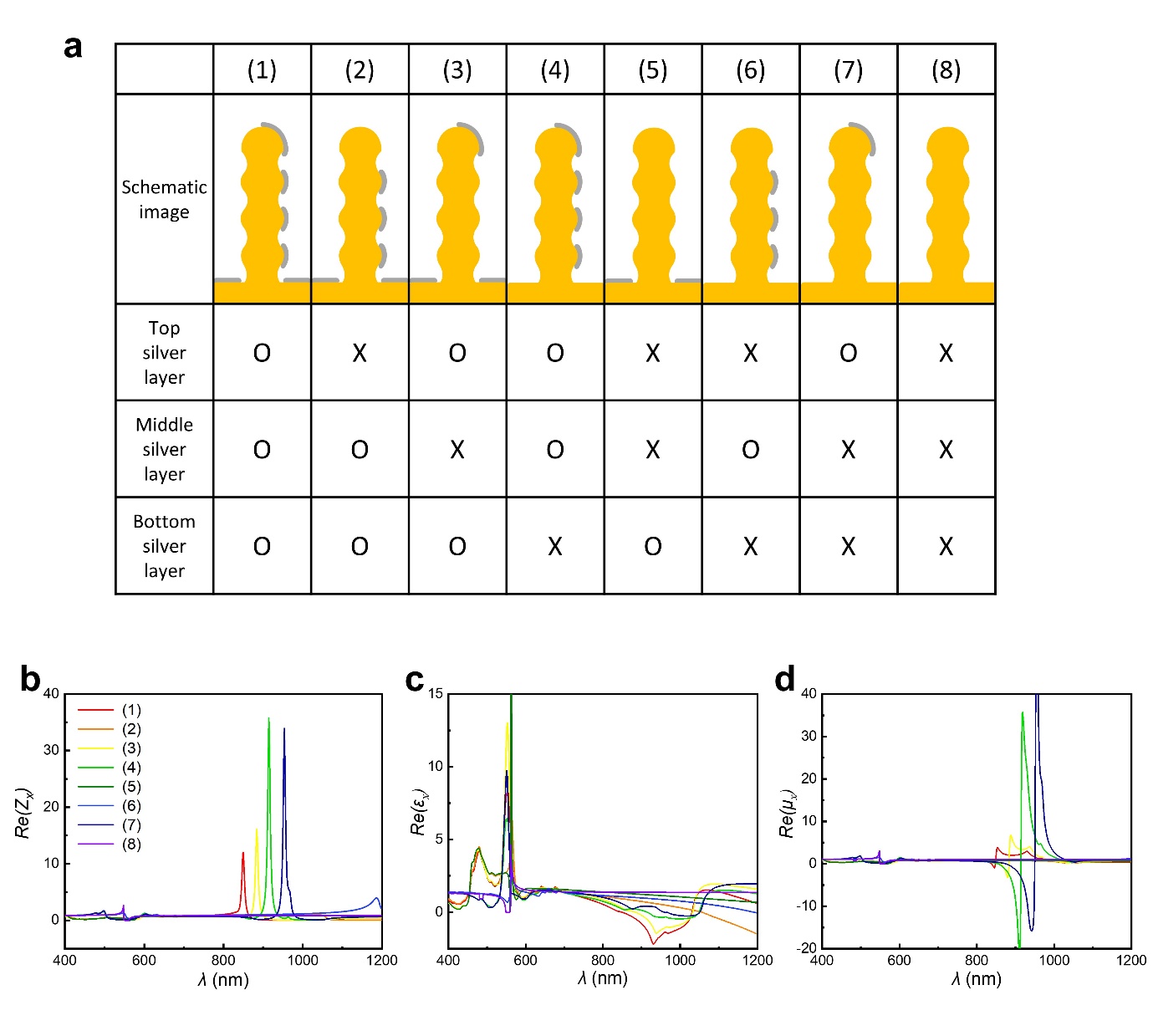
**

**Figure S7**. Effective optical parameters (Z, ε, and μ) of the SSRR substructure come from S-parameter retrieval method. (a) Schematic image and inclusion relationship table for each substructure. (b) Effective wave impedance, (c) permittivity, and (d) permeability under x-polarized light.

**S8. Geometric size effects of SSRR on the reflectance differences**

**
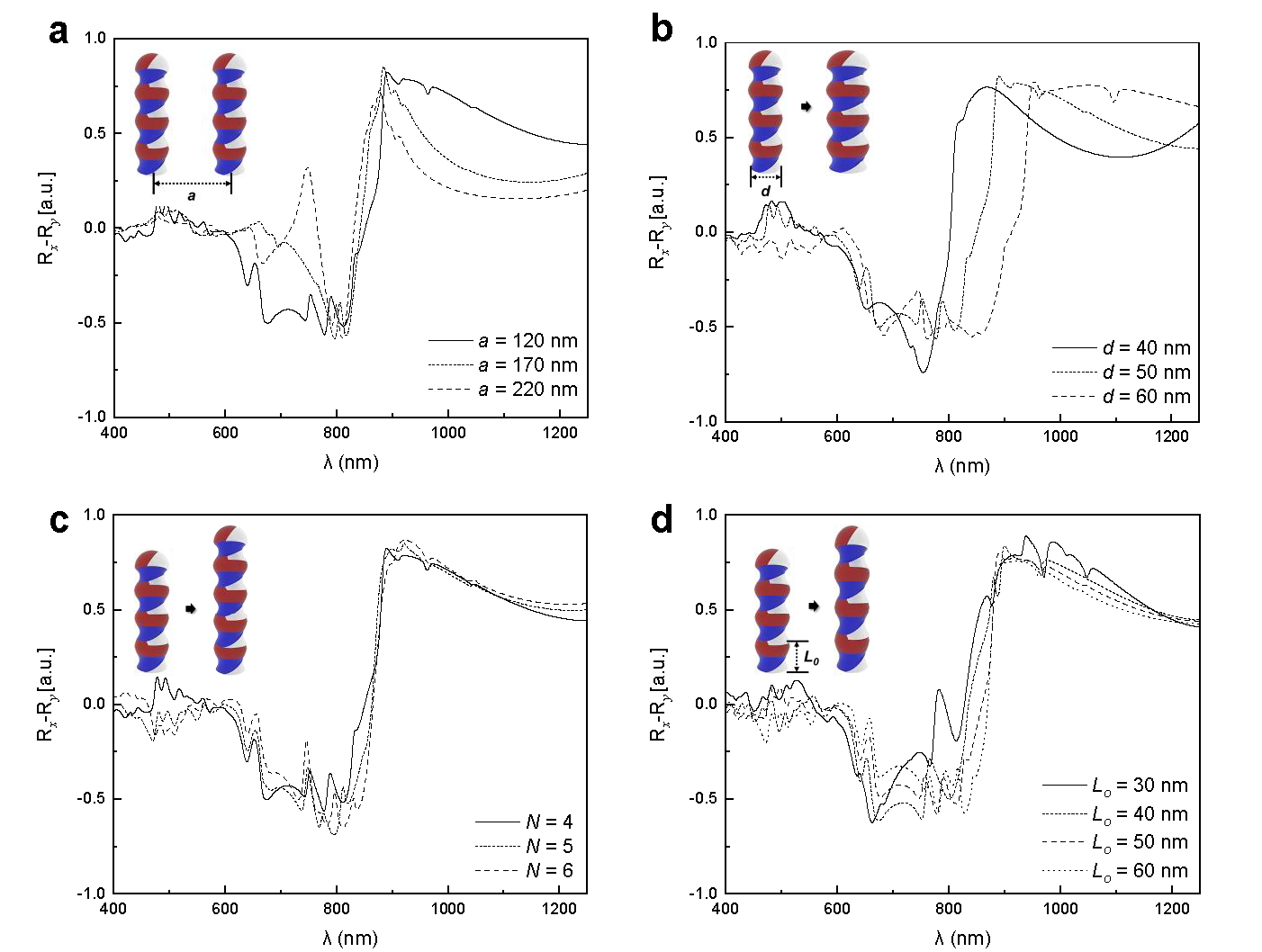
**

**Figure S8.** Effect of (a) the center-to-center distance between nanorods *a,* (b) the nanorod diameter *d*, (c) the number of ring *N*, and (d) neighboring inter-rod distance *L_o_* on the reflectance differences between *x*- and *y*-polarized light (R_x_-R*_y_*).

**S9 A low-density array of SSRR nanorods with large periodicity.**


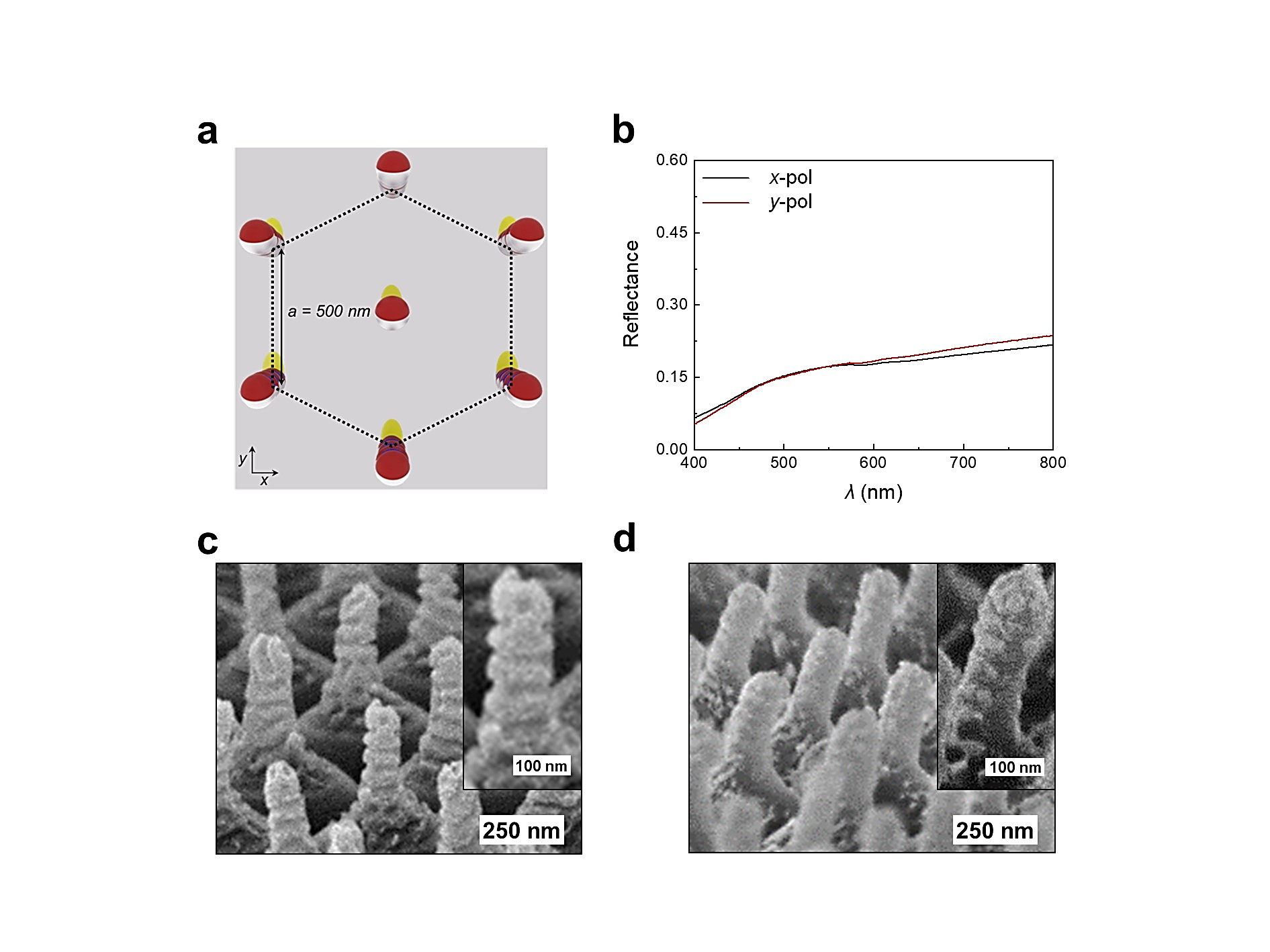


**Figure S9** (a) Schematic image of a hexagonal array of SSRR (a: the center-to-center distance between two neighboring nanorods). (b) Experimentally measured reflectance of the array of SSRR**.** FE-SEM image of a low-density array of (c) pagoda-like nanorods (d) SSRR with large perodicity (a = 500 nm)
